# Harnessing the Intradermal Delivery of Hair Follicle Dermal Papilla Cell Spheroids for Hair Follicle Regeneration in Nude Mice

**DOI:** 10.34133/bmr.0129

**Published:** 2025-01-13

**Authors:** Moon Sung Kang, Mina Kwon, Rowoon Park, Jaeheung Kim, Suck Won Hong, Chang-Seok Kim, Won Jun Yang, Ki Su Kim, Dong-Wook Han

**Affiliations:** ^1^ Research Institute of Mechanical Technology, Pusan National University, Busan 46241, Republic of Korea.; ^2^ School of Chemical Engineering, Pusan National University, Busan 46241, Republic of Korea.; ^3^ Department of Cogno-Mechatronics Engineering, Pusan National University, Busan 46241, Republic of Korea.; ^4^ Engineering Research Center for Color-Modulated Extra-Sensory Perception Technology, Pusan National University, Busan 46241, Republic of Korea.; ^5^ SUNIN CNS, Seoul 13453, Republic of Korea.; ^6^ Institute of Advanced Organic Materials and Department of Organic Materials Science and Engineering, Pusan National University, Busan 46241, Republic of Korea.; ^7^ Institute of Nano-Bio Convergence, Pusan National University, Busan 46241, Republic of Korea.

## Introduction

Hair loss, particularly androgenetic alopecia (AGA), the most common type of male-pattern baldness, is a prevalent concern in today’s society, impacting physical appearance and psychological conditions. Although various treatments including chemical drugs, herbal extracts, platelet-rich plasma, and hair transplantation are available, many have yielded limited success and long-term efficiency [1]. Recent advancements in cell therapy, tissue engineering, and regenerative medicine have provided new hope for more effective hair loss treatments. However, delivery of hair follicle (HF) stem cells to humans has been limited by the loss of trichogenic potential in the transplanted cells and suboptimal culture conditions [2]. HF dermal papilla cells (HFDPCs), composed of mesenchymal cells in HFs, play a crucial role in HF embryogenesis and hair growth cycle regulation. Therefore, maintaining the survival and hair inductivity of HFDPCs both in the in vitro and in vivo settings is critical for potential clinical applications. In this study, we introduce a robust protocol to fabricate small-sized (50 to 70 μm) HFDPC spheroids while maintaining high cell viability and hair inductivity. The intradermal injection of HFDPC spheroids induced substantial hair shaft formation in nude mice, demonstrating the potential of the therapy for future hair loss treatments.

## Materials and Methods

### Experimental design

**Fig. 1.**
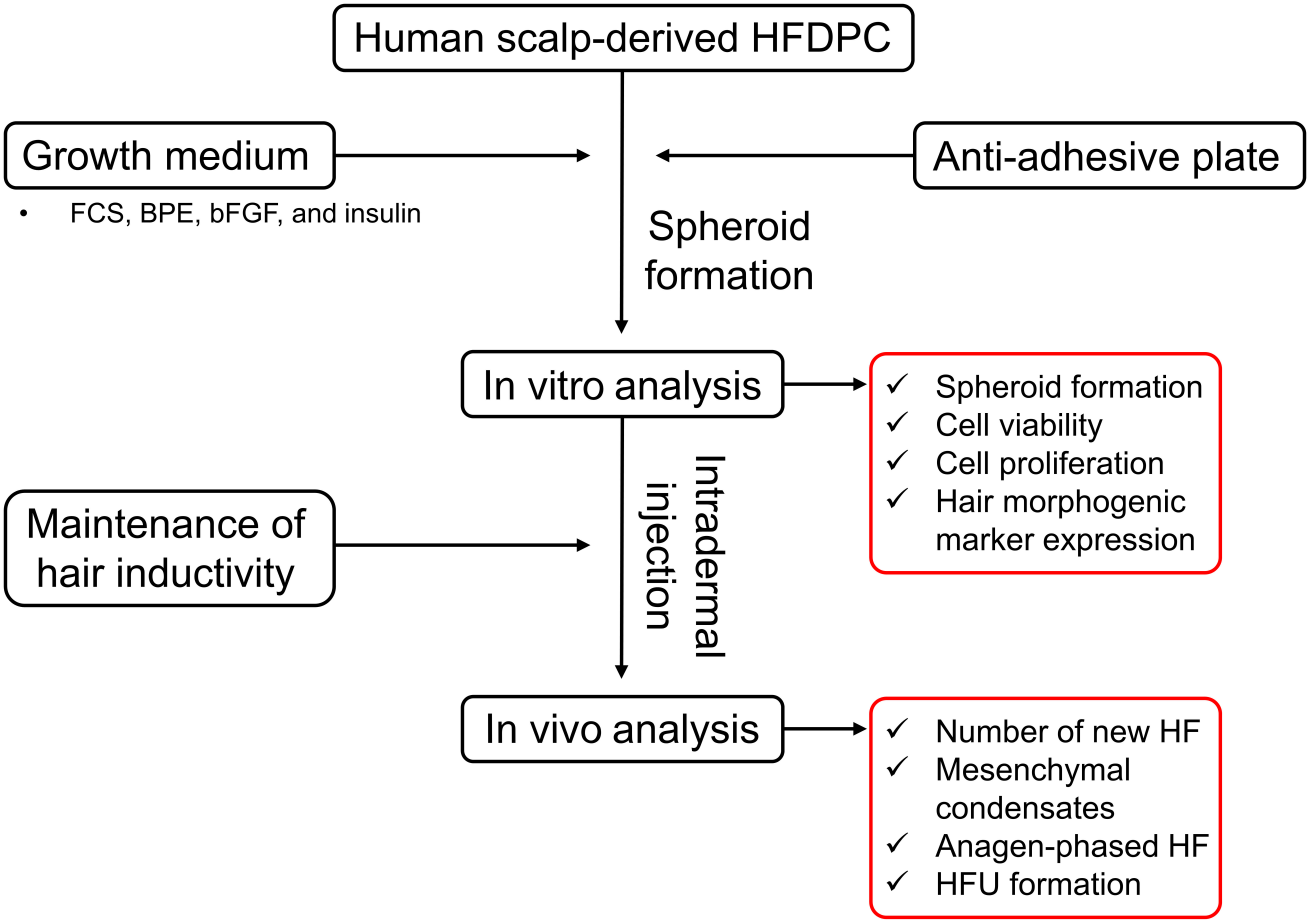
Flowchart of the entire experimental design. HFDPC, hair follicle dermal papilla cell; FCS, fetal calf serum; bFGF, basic fibroblast growth factor; HF, hair follicle; HFU, hair follicle unit.

This study aims to intradermally deliver HFDPC spheroids into BALB/c nude mice to induce neogenesis of the hair shafts. The human scalp dermis-derived HFDPC spheroids were cultured in the optimum in vitro conditions and delivered to the dorsal skins of mice as depicted in Fig. 1.

### Cell culture conditions and spheroid fabrication

The primary HFDPCs from the human scalp dermis were purchased from PromoCell (Heidelberg, Germany). HFDPCs were cultured using a basal growth medium supplemented with fetal calf serum (FCS) at a concentration of 0.04 ml/ml, bovine pituitary extract (BPE) at 0.004 ml/ml, basic fibroblast growth factor (bFGF) at 1 ng/ml, and insulin at 5 μg/ml. This medium was supplemented with a 1% (v/v) antibiotic–antimycotic solution, comprising 10,000 units/ml penicillin, 10 mg/ml streptomycin, and 25 μg/ml amphotericin B per (Sigma-Aldrich, St. Louis, MO). HFDPCs from passages 2 to 3 were detached using trypsin-EDTA (Welgene, Daegu, Republic of Korea), and cells at a density of 10^5^ cells/ml were seeded onto anti-adhesive culture plates (Labtolab, Daejeon, Republic of Korea). Subsequently, they were cultured using the HFDPC growth medium for predetermined periods. As a control, murine fibroblast L929 cell lines were cultured using a Dulbecco’s modified Eagle’s medium (DMEM) supplemented with 10% (v/v) fetal bovine serum (FBS) and 1% (v/v) antibiotic–antimycotic solution.

### ALP staining of HFDPC spheroids

Alkaline phosphatase (ALP) staining of HFDPC spheroids was conducted using the 1-Step NBT (nitro blue tetrazolium)/BCIP (bromochloroindolyl phosphate) solution (Sigma-Aldrich). After 14 d of culture, HFDPC spheroids were collected in a 15-ml tube and centrifuged for 4 min at 1,200 rpm. The spheroid pellets were prepared by removing the supernatant and homogeneously mixed with NBT/BCIP solution, and subsequently maintained at 25 °C for predetermined periods. Images of the stained spheroids were captured using an optical microscope (IX81, Olympus, Tokyo, Japan).

### Animal test conditions and intradermal delivery of HFDPC spheroids

The Animal Care Committee of Pusan National University (PNU-2023-0284) approved all animal procedures. Male BALB/c nude mice, aged 8 weeks, were housed in a specific pathogen-free (SPF)-rated barrier animal room. Mice were sedated with 3% isoflurane and maintained under anesthesia with 1% to 1.5% isoflurane. Approximately 100 to 150 spheroids were suspended in sterilized 10-μl Dulbecco’s phosphate-buffered saline (DPBS) and intradermally injected into the dorsal skin of mice.

### Histological analysis

For skin histology, 10-μm paraffin-embedded sections were collected and stained with hematoxylin and eosin (H&E; Abcam) dyes according to the manufacturer’s instructions. After staining, images were captured using an AxioScan Z1 digital slide scanner (Zeiss). Images were analyzed using Zen Blue software (Zeiss). Quantitative data such as mesenchymal condensate, anagen follicle, and hair follicle unit (HFU) were obtained from the H&E images using ImageJ software.

### Statistical analysis

All variables were tested in 3 independent experiments, each performed in duplicate on 3 different cultures (*n* = 6). Data are presented as mean ± SD. Data were tested for equality of variances using Levene’s test before statistical analysis. Statistical multiple comparisons were performed using the Bonferroni test after preliminary analysis of variance (ANOVA). Asterisks (* to ****) indicate statistical significance (*P < 0.05, **P < 0.01, ***P < 0.001, and ****P < 0.0001).

## Results

### Cell viability and proliferation of HFDPC spheroids

**Fig. 2.**
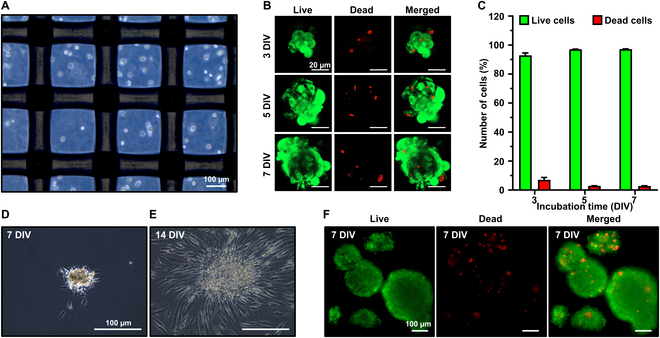
Fabrication of HFDPC spheroids and cell viability evaluation. (A) Optical microscopy immediately after seeding. (B) Live/dead assay and (C) the ratio of live and dead cells at 3, 5, and 7 DIV. HFDPC spheroids cultured on TCP at (D) 7 DIV and (E) 14 DIV. (F) Live/dead assay on the HFDPC spheroids prepared by hanging drop.

The routinely cultured HFDPCs were seeded on the latticed anti-adhesive plates, and the prepared growth media (see details in Materials and Methods) were added to facilitate the growth of spheroids (Fig. 2A). The cell viability of HFDPC spheroids was evaluated by live/dead assay (Fig. 2B and C). On 3 days in vitro (DIV), the HFDPCs formed premature spherical morphology with high cell viability (92.95% viable). On 5 and 7 DIV, the cells underwent constant proliferation to increase the diameter and cell population to form a mature spheroid. Additionally, the ratio of dead cells was gradually decreased (2.87% at 5 DIV and 2.76% at 7 DIV). To further monitor the maintenance of spreading and proliferation in HFDPC spheroids, they were transferred to tissue culture plastics (TCPs), where the spheroids progressively increased in size, and individual cells spread across the TCPs on 14 DIV (Fig. 2D and E). Compared to the widely used spheroid fabrication method (i.e., hanging drop technique) [3], the prepared HFDPC spheroids on anti-adhesive lattices maintained consistently small sizes and exhibited high viability (Fig. 2F).

**Fig. 3.**
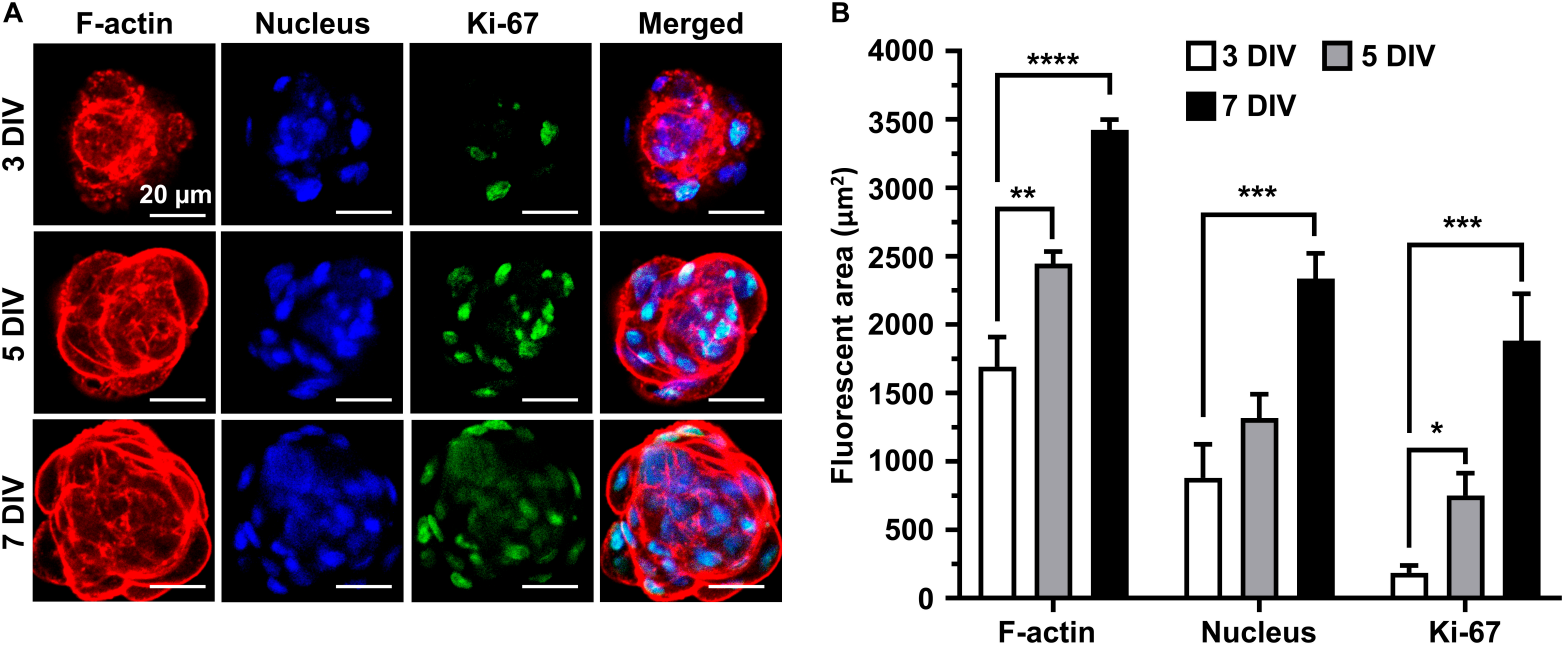
Immunocytochemical analysis of HFDPC spheroids. (A) Fluorescence microscopy on HFDPC spheroids on 3, 5, and 7 DIV. (B) Quantitative analysis of fluorescence intensity on TRITC (F-actin), DAPI (nucleus), and fluorescein isothiocyanate (FITC) (Ki-67). The asterisks indicate significant differences between groups (*P < 0.05, **P < 0.01, ***P < 0.001, and ****P < 0.0001).

Figure 3A and B shows that the characteristic F-actin condensation was observed on 3 to 7 DIV, and the HFDPC spheroid exhibited round morphology. The fluorescence intensities from tetramethylrhodamine isothiocyanate (TRITC) and 4’,6-diamidino-2-phenylindole (DAPI) were gradually increased, along with the elevated expressions of Ki-67^+^ nuclei, indicating that the proliferation of HFDPC spheroids remained active during 7 DIV. Additionally, due to the relatively small sizes (50 to 70 μm), the HFDPC spheroids showed highly proliferative nuclei in the core by avoiding hypoxia-mediated necrosis [4].

### Hair inductivity of HFDPC spheroids in vitro

**Fig. 4.**
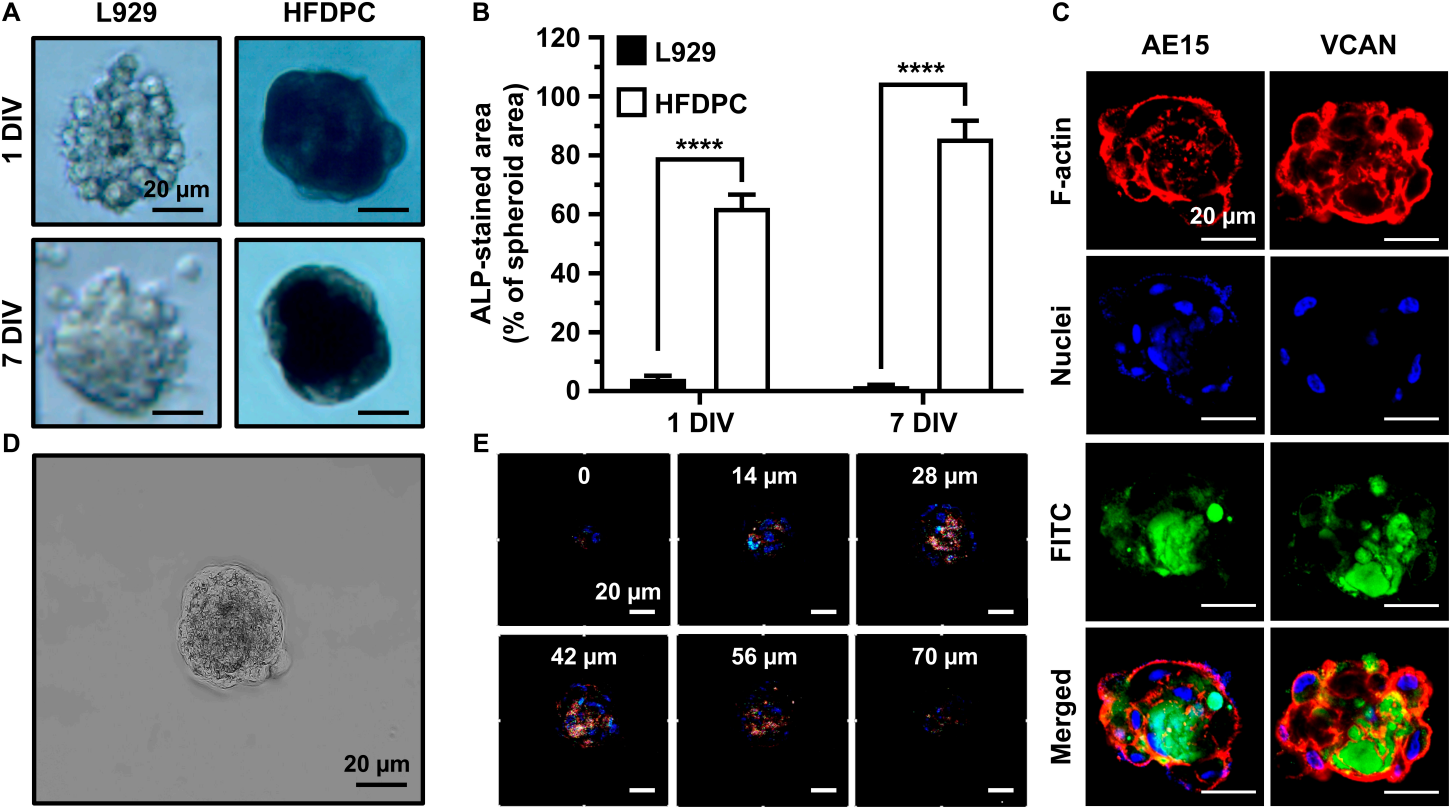
Hair inductivity of HFDPC spheroids. L929 spheroids were used as control groups. (A) ALP staining of L929 and HFDPC spheroids on 1 and 7 DIV. (B) Quantification of ALP-stained area on 1 and 7 DIV. (C) Immunofluorescence staining of HFDPC spheroids on 7 DIV. FITC labels AE15 and VCAN. (D) Optical microscopy of HFDPC spheroid on 7 DIV. (E) Two-photon microscopy with a depth profiling of 0 to 70 μm. Fluorescence channels are indicated as follows: blue, nucleus; red, F-actin; green, VCAN. The asterisks indicate significant differences between groups (****P < 0.0001).

We further evaluated whether the hair inductivity of HFDPC spheroids remained intact in the in vitro environment by assessing the expression of ALP, AE15 (trichohyalin), and VCAN (versican). On both 1 and 7 DIV, HFDPC spheroids exhibited distinct condensed cytoskeletal structures along with the larger area of ALP staining (dark stained area), whereas L929 cells formed immature spheroids without any ALP-stained area (Fig. 4A and B). On 7 DIV, HFDPC spheroids exhibited clear expression of AE15 and VCAN, which are characteristic markers of hair-inducible anagen HFs (Fig. 4C) [5,6]. Depth-profiling images using 2-photon microscopy showed that the inner core of HFDPC spheroids exhibited higher VCAN expression (green fluorescence) compared to that of the surface (Fig. 4D and E).

### In vivo intradermal delivery of HFDPC spheroids into dorsal skin of nude mice

**Fig. 5.**
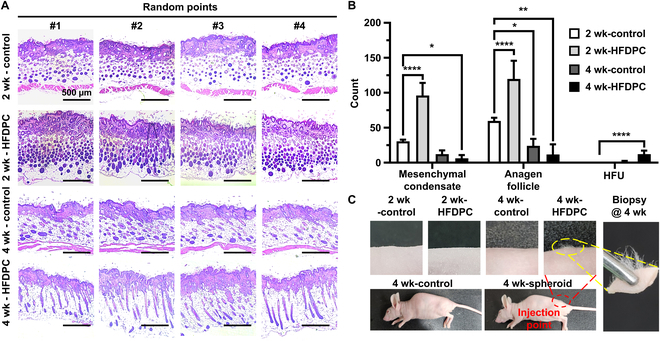
Intradermal delivery of HFDPC spheroids into nude mice. (A) H&E-stained images and (B) quantitative analysis on the number of mesenchymal condensates, anagen follicles, and HFUs. (C) Digital images of dorsal skins of sacrificed mice at 2 and 4 weeks after injection. Red and yellow dotted lines denote the injection point and biopsy area, respectively.

The prepared HFDPC spheroids were loaded in the syringe and delivered into the dorsal skin of nude mice by intradermal injection. After 2 weeks, HFDPC spheroid-injected groups showed an increased number of mesenchymal condensates (core-shell-like morphology), which are the buds for HF neogenesis, compared to the control (DPBS-injected) (Fig. 5A and B) [7]. In addition, the number of anagen-phased follicles (cell-filled HFs) was significantly increased in the HFDPC spheroid-injected group, while most HFs in the DPBS groups remained terminal anagen morphology (empty cells) [8]. On 4 weeks after injection, the increased number of mesenchymal condensates and anagen follicles ultimately led to a greater number of vertically elongated HFU. Notably, in the groups injected with HFDPC, new hair shafts emerged, protruding from the dorsal skin and exhibiting voluminous white hair (Fig. 5C), suggesting that the intradermal delivery of HFDPC spheroids can induce hair regeneration in the in vivo environment.

## Discussion

In this study, we demonstrated that intradermal delivery of in vitro-cultured HFDPC spheroids can promote new hair shaft formation. Recent studies have highlighted specific methods to fabricate HFDPC spheroids. Compared to these methods, our aggrewell-based method has distinct advantages such as high synthesis yields, size uniformity, and small sizes in a controllable manner. As a result, our HFDPC spheroids could sustain high cell viability and strong expression of hair-inductive markers, comparable to or even exceeding those introduced by previous methods (Table S1).

Due to their relatively small size (50 to 70 μm), the spheroids sustained high cell viability within their cores by preventing hypoxic conditions. HFDPC cells have a doubling time of 20 to 36 h, leading to a 128-fold increase in cell count and a 5.04-fold increase in spheroid volume and diameter. Although this calculation does not fully replicate intercellular complexities in the 3-dimensional niche, our results correlate to this prediction (50 to 70 μm on 1 DIV and 250 to 300 μm on 7 DIV). Thus, an optimal spheroid size of 50 to 70 μm was set to maintain high cell viability between 7 DIV. As HFDPC spheroids grow larger, cell proliferation in the core stops and initiates hair morphogenesis [9]. Given that our spheroids are relatively small, they remained stable and continued proliferating within the core, keeping them in optimal condition before transplantation. Additionally, the high expression of AE15 and VCAN demonstrates that the prepared HFDPC spheroids closely resemble the primitive inner root sheath and the condensed mesenchyme during HF embryogenesis [5,10]. The ALP is widely expressed in actively proliferating or remodeling tissues to support phosphate metabolism and the regulation of ions and biomolecules. HFs in the anagen phase are highly regenerative, and the early passage of HFDPCs with ALP activity is known to induce HF formation, as observed in our results [11]. As a result, intradermal injection of HFDPCs into nude mice induced an increased number of mesenchymal condensates, anagen follicles, and HFUs, which finally induced voluminous hair shaft protrusion.

AGA patients often exhibit overexpression of insulin-like growth factor 1 (IGF-1), which inhibits the mitogen-activated protein kinase (MAPK) signaling pathways, resulting in the shrinkage of HFs and blood vessels. Transplanting intact HFDPC spheroids is anticipated to trigger paracrine effects to up-regulate MAPK pathways [12]. Furthermore, transplanting HFDPC spheroids can act as seeds for new HF organogenesis, potentially leading to the formation of new hair shafts [13]. Additionally, long-term survival and potential side effect issues (e.g., colonization and blood vessel clotting) of injected HFDPC spheroids are critical concerns [14]. Future studies would focus on clarifying the rationales behind the hair growth effects by examining the origin of active HFs and the structural integrity and functionalities of new HF organelles. Furthermore, the long-term stability of injected HFDPC spheroids should be assessed through extensive preclinical testing. HFDPC delivery faces challenges with immune rejection, especially when using allogeneic cells, which often trigger host immune responses, leading to potential graft rejection. Strategies including immunosuppression and human leukocyte antigen matching can be applied to mitigate this, but understanding specific immune mechanisms is essential to improve long-term graft survival and clinical success [15].

In conclusion, the following studies and extensive clinical tests are required to unveil the long-term survival and potential safety issues, as well as an in-depth biological analysis of the reliability and efficiency. These limitations highlight the areas for future research and development to improve the effectiveness and applicability of cell therapy for hair loss therapies.

## Data Availability

All data relevant to this work are presented within the article and accompanying supplementary material.

## Supplementary Materials

Supplementary 1 Supplementary Note Table S1 References [16–20]
