# Supplementary material for: Harnessing the Intradermal Delivery of Hair Follicle Dermal Papilla Cell Spheroids for Hair Follicle Regeneration in Nude Mice
Source: Biomater Res. 2025 Jan 13;29:0129. doi: 10.34133/bmr.0129 (PMC11725629; doi:10.34133/bmr.0129)
Supplement: Supplementary 1 — Supplementary Note Table S1 References [16–20] [file bmr.0129.f1.doc]

***A. Supplementary note***

**Live/dead assay and immunofluorescence staining of HFDPC spheroids**

The HFDPC spheroids were collected and centrifuged at 1200 rpm for 4 min in a 15 mL tube, with the sterilized Dulbecco’s phosphate-buffered saline (DPBS) washing repeated for each subsequent step. After removing the supernatant, the spheroid pellets were homogeneously mixed with 2 µM calcein AM (C1430) and 4 µM ethidium homodimer-1 (E1169) (Invitrogen, Waltham, MA). After 30 min of incubation in the 37°C incubator at dark, the spheroids were moved to 6-well plates and briefly rinsed with DPBS to remove the unreacted dyes. For immunofluorescences staining, the spheroids were treated with 3.7% formaldehyde solution (Sigma-Aldrich) for 10 min, followed by 0.1% Triton X-100 (Sigma-Aldrich) for 5 min and blocking with 2% bovine serum albumin (BSA, Sigma-Aldrich) for 30 min. Anti-versican (VCAN) (ab19345, Abcam, Cambridge, UK), anti-AE15 (ab58755, Abcam), and anti-Ki67 (ab16667, Abcam) antibodies were then added and reacted for 24 h at 4°C. Subsequently, fluorescein isothiocyanate (FITC)-labeled second antibodies were applied for 1 h. Additionally, 165 nM tetramethylrhodamine isothiocyanate (TRITC)-labeled phalloidin (R415, Thermofisher Scientific, Waltham, MA) and 300 nM 4',6-diamidino-2-phenylindole (DAPI, D1306, Thermofisher Scientific) solution were added and incubated for 30 min at 25°C in the dark. Fluorescence images were captured using CLSM (LSM 800, Zeiss, Oberkochen, Germany) and custom-built two-photon microscopy [16]. To assess the ratio of live and dead cells, we captured several layers above and below the focal plane by z-stacking mode and repeated manual counting to minimize the errors (n=6).

***B. Supplementary table***

**Table S1.** Comparison of the characteristics of HFDPC spheroids from this study with those from previous studies.

| **Size (μm)** | **Cell viability (%)** | ***In vitro* hair-inductive marker expression** | ***In vivo* hair-inductive marker expression** | **Ref.** |
| --- | --- | --- | --- | --- |
| 50 – 70 | 93 – 97 | - ALP, VCAN, and AE15 | - Anagen follicle, mesenchymal condensate, and HFU  - Hair shafts formation | This study |
| 76 – 121 | NA | - K14, K5, K40, LEF1, GATA3, and SOX9  - Hair shaft formation | NA | [9] |
| NA | NA | - ALP, Wnt, BMP, and FGF signaling markers  - ID2, SOX2, and HEY1 | NA | [17] |
| NA | NA | - Gene profiling on growth-related and immune system-related pathways | NA | [18] |
| ≈ 200 | > 90 | - ALP, fibronectin, VCAN, vimentin | HF neogenesis | [19] |
| NA | NA | - DP signature transcriptome on hair inductivity | ALP, inner root sheaths, and hair shafts. | [20] |
